# A 25-Year History of Leg Ulceration: Cutaneous Blastomycosis

**DOI:** 10.5826/dpc.1003a54

**Published:** 2020-06-29

**Authors:** Ömer Faruk Elmas, Belkiz Uyar, Asuman Kilitçi

**Affiliations:** 1 Department of Dermatology, Faculty of Medicine, Kırşehir Ahi Evran University, Kırşehir, Turkey; 2 Department of Pathology, Faculty of Medicine, Kırşehir Ahi Evran University, Kırşehir, Turkey

**Keywords:** cutaneous blastomycosis, dermoscopy, histopathology, pyoderma gangrenosum

## Introduction

Blastomycosis is a fungal infection caused by *Blastomyces dermatitidis*. It is characterized by a chronic granulomatous and suppurative inflammatory reaction [1]. Cutaneous blastomycosis (CB) usually develops after hematogenous dissemination from primary lung involvement. However, less commonly, *Blastomyces dermatitidis* can be inoculated into the skin, causing primary cutaneous blastomycosis. The skin involvement in CB includes a wide variety of manifestations ranging from papulopustular and nodular lesions to verrucous and ulcerative lesions [2]. We report a case of CB initially misdiagnosed as pyoderma gangrenosum (PG).

## Case Presentation

**Figure 1:**
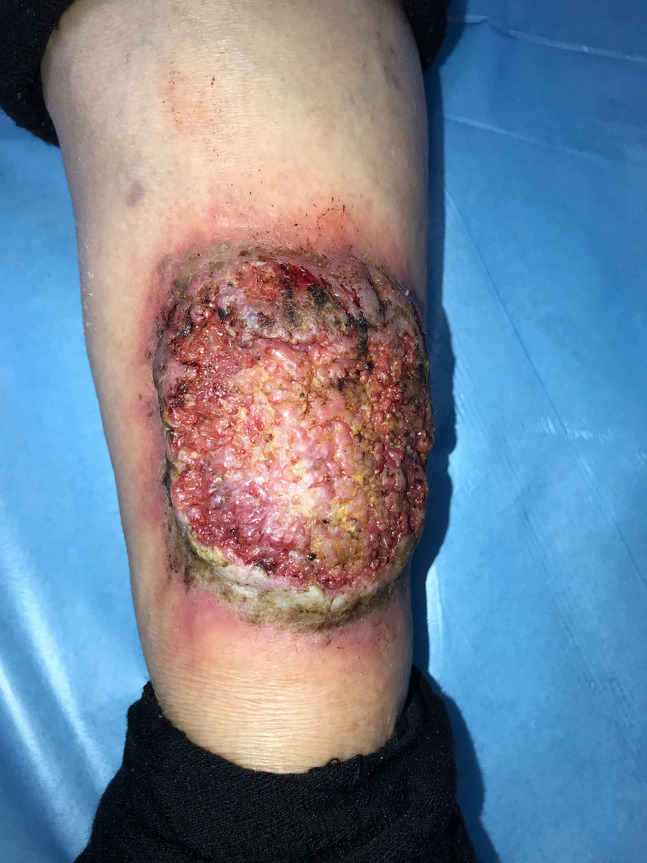
Vegetative ulceration 18 × 10 cm in size on anterior aspect of the left leg.

**Figure 2:**
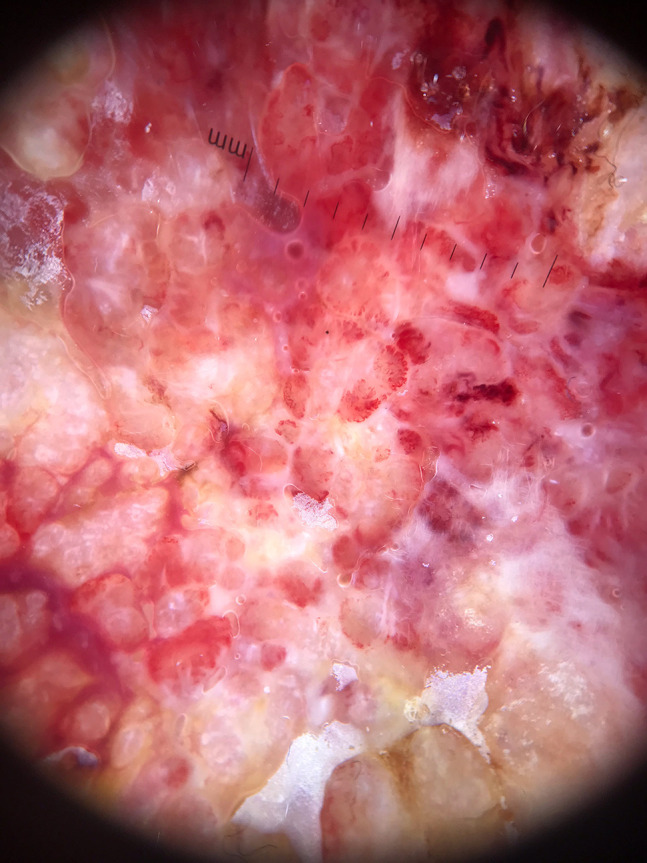
Dermoscopy shows white to pinkish overlapping papillomatous structures, white and red structureless areas, blood spots, and polymorphous vessels including dotted, coiled, serpentine complex looped vessels with an annular arrangement.

**Figure 3:**
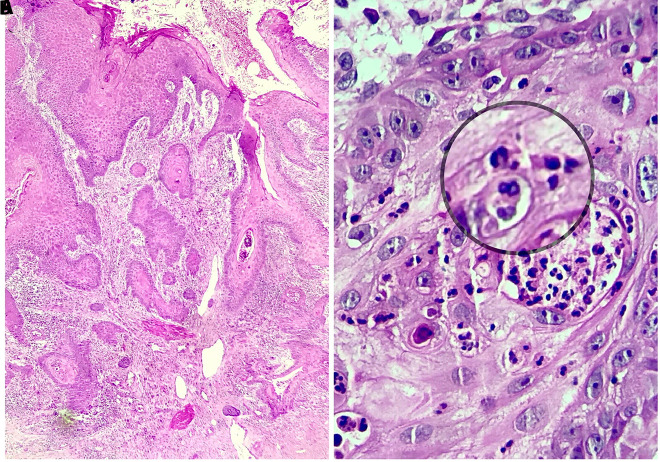
(A) Low-power histopathological view shows ulceration, pseudoepitheliomatous hyperplasia, and dermal inflammatory infiltration (H&E, ×100). (B) Broad-based bugging yeasts consistent with *Blastomyces dermatitidis* (H&E, ×400).

A 64-year-old woman with a nonhealing ulceration on the left leg was referred to us with a preliminary diagnosis of PG. The lesion first appeared 25 years ago and had grown in size without any response to many treatments, including topical and systemic antibiotics and corticosteroids. There was no history of a trauma to the area or a known inciting cause. There was also no history of a respiratory infection. Dermatological examination revealed a painless vegetative ulceration 18 × 10 cm in size on the anterior aspect of the left leg (Figure 1). On the dermoscopic examination, white to pinkish overlapping papillomatous structures, white and red structureless areas, blood spots, and polymorphous vessels including dotted, coiled, serpentine, and complex looped vessels with an annular arrangement were observed (Figure 2). Direct microscopic examination of the skin-scraping material revealed the fungal element. The histopathological examination showed ulceration, pseudoepitheliomatous hyperplasia, predominantly neutrophilic inflammatory infiltrate, and broad-based bugging yeasts consistent with *Blastomyces dermatitidis* (Figure 3). A detailed workup revealed no evidence of systemic involvement. A 200-mg daily dose of itraconazole was started; considerable improvement was observed after 3 months.

## Conclusions

CB is a rare and often misdiagnosed cause of persistent leg ulceration. The common misdiagnoses include scrofuloderma, granuloma inguinale, nocardiosis, cutaneous tuberculosis, as well as squamous cell carcinoma. Our patient was initially misdiagnosed with PG and underwent systemic corticosteroid treatment, which did not lead to remarkable improvement. PG is a neutrophilic dermatosis characterized by painful ulcerative lesions usually located on extremities. On histopathological examination, PG usually shows ulceration, pseudoepitheliomatous hyperplasia, and neutrophil-rich dermal inflammatory infiltration, which can also be seen in CB. The significant clinicopathological overlap between CB and PG may cause misdiagnosis. The diagnosis of recalcitrant leg ulcerations including PG is mainly based on the exclusion and histopathological examination is usually performed. In this context, pathology specimens submitted should always be examined carefully to rule out CB. It should also be kept in mind that in a case of CB, the misdiagnosis of PG followed by systemic corticosteroid therapy may cause fatal complications such as disseminated blastomycosis and acute respiratory distress syndrome [2,3].
